# Uncertainty affects cancer-related fatigue among breast cancer women undergoing peripherally inserted central catheter chemotherapy: the chain mediating role of psychological resilience and self-care

**DOI:** 10.1186/s12905-024-03187-9

**Published:** 2024-06-15

**Authors:** Yue Yang, Shihui Liu

**Affiliations:** 1 Department of Hepatobiliary and Pancreatic Surgery, First Hospital of China Medical University, No. 210, Baeta 1 Street, Hunnan District, Shenyang, Liaoning 110001 China; 2 Operating Room, First Hospital of China Medical University, No. 155 Nanjing North Street, Heping District, Shenyang, Liaoning 110001 China

**Keywords:** Breast cancer, Cancer-related fatigue, Chain mediating effect, PICC chemotherapy, Psychological resilience, Self-care, Uncertainty

## Abstract

**Background:**

Breast cancer patients undergoing chemotherapy via peripherally inserted central catheter often experience serious behavioral and psychological challenges, with uncertainty and cancer-related fatigue being prevalent issues that profoundly impact prognosis. Therefore, this study aimed to investigate the relationship between uncertainty and cancer-related fatigue by employing a chain mediation model to examine the potential mediating roles of psychological resilience and self-care.

**Methods:**

A cross-sectional study was conducted with 223 breast cancer patients receiving peripherally inserted central catheter chemotherapy at two tertiary affiliated hospitals of China Medical University in Liaoning, China, from February 2021 to December 2022. Participants completed self-reported questionnaires to assess uncertainty, psychological resilience, self-care, and cancer-related fatigue. The collected data were subsequently analyzed using Pearson’s correlation analysis, hierarchical regression analysis, and mediation analysis.

**Results:**

Uncertainty exhibited a significant positive correlation with cancer-related fatigue (*p* < 0.01) and a negative correlation with psychological resilience (*p* < 0.01) and self-care (*p* < 0.01). Uncertainty was found to impact cancer-related fatigue through three pathways: psychological resilience mediated the relationship between uncertainty and cancer-related fatigue (mediating effect = 0.240, 95% confidence interval: 0.188 to 0.298, effect ratio = 53.22%); self-care also mediated this relationship (mediating effect = 0.080, 95% confidence interval: 0.044 to 0.121, effect ratio = 17.74%); furthermore, there was a significant joint mediating effect of psychological resilience and self-care on the association between uncertainty and cancer-related fatigue (mediating effect = 0.042, 95% confidence interval: 0.021 to 0.068, effect ratio o = 9.31%).

**Conclusion:**

The findings of this study revealed that uncertainty not only directly influenced cancer-related fatigue, but also operated through the mediating effect of psychological resilience, self-care, and sequential mediation of psychological resilience and self-care. Interventions tailored for breast cancer patients receiving peripherally inserted central catheter chemotherapy should target these factors to help alleviate uncertainty, enhance psychological resilience, and improve self-care practices, thereby ameliorating cancer-related fatigue.

## Introduction

Breast cancer (BC) is the most common malignancy among women globally, with approximately 4.8 million new cases and 3.2 million deaths reported in China in 2022 [1]. Adjuvant chemotherapy stands as a primary treatment strategy for BC in clinical practice, with intravenous infusion via a peripherally inserted central catheter (PICC) increasingly serving as the primary method for long-term administration of chemotherapeutic agents and parenteral nutrition [2]. Nevertheless, the extended use of PICC insertion for delivering chemotherapeutic drugs has been linked to various psychological burdens in cancer patients [3], with cancer-related fatigue (CRF) and cancer-related uncertainty being deemed the primary adverse outcomes of chemotherapy for BC patients [4].

## Research theory and hypothesis

### Hypothesis 1: uncertainty positively and directly affects CRF

CRF manifests as subjective feeling of persistent tiredness that exerts diverse detrimental effects on the cognitive and mental functions of cancer survivors in their daily lives [5]. Individuals experiencing CRF often exhibit symptoms such as generalized physical weakness, reduced social engagement, impaired attention or concentration, significant emotional distress, and excessive sleepiness that persists even after periods of rest [6]. Previous literature has indicated that about 70–100% of BC patients undergoing chemotherapy treatment will encounter moderate to severe CRF symptoms, with the severity of CRF significantly higher in these patients compared to those not undergoing chemotherapy [7]. Follow-up studies conducted in clinical settings have revealed that nearly one-third of cancer survivors can experience persistent fatigue up to 6 years post-treatment completion [8]. Consequently, CRF adversely impacts the physical and psychological health of patients, ultimately deteriorating their health-related quality of life and potentially shortening their survival span [9]. Despite efforts to mitigate the debilitating effects of CRF on patient prognosis through various interventions [10], a substantial gap remains in understanding the underlying mechanisms of CRF in BC patients receiving PICC chemotherapy.

The lack of information and experience among cancer patients often leads to cancer-related uncertainty during their prolonged periods of radiotherapy and chemotherapy, impacting treatment adherence, physical well-being, and mental adjustment [11]. Cancer-related uncertainty is defined as confusion and ambiguity concerning cancer symptoms, treatment side effects, sequelae duration, and fear of recurrence [12]. In BC patients, the severity of disease uncertainty about the disease is closely associated with increased levels of anxiety, depression, and fatigue, directly influencing prognosis and quality of life [13]. Zhang et al. observed significant uncertainty among Chinese BC patients undergoing chemotherapy due to their limited familiarity with complex cancer treatment [14]. Furthermore, prior study has verified a positive correlation between cancer-related uncertainty and CRF in older BC survivors [15]. Therefore, BC patients with heightened CRF concerns might benefit from uncertainty management strategies. Understanding the regulatory mechanism linking cancer-related uncertainty and CRF in BC patients undergoing PICC chemotherapy is essential for effective management development.

### Hypothesis 2: psychological resilience plays a mediating role in the association between uncertainty and CRF

In addition to the aforementioned adverse psychological aspects, it is noteworthy that BC survivors also undergo positive changes during their therapeutic periods. Psychological resilience refers to an individual’s perception of their ability to effectively withstand stressful circumstances, gradually adapt, and recover from the challenging situations. Moreover, a high level of psychological resilience has been shown to enhance the quality of life in BC patients by fostering beneficial emotions like self-esteem, positive moods, and optimistic attitudes [16]. Therefore, boosting psychological resilience is essential for BC patients not only to improve their physical and psychological outcomes, but also to facilitate adaptation to lifestyle medications in the face of malignant conditions [17]. Evidence also indicates that patients exhibiting high levels of psychological resilience are better equipped to cope with negative disease experiences including anxiety, depression, and CRF among Chinese patients with gastric and colon cancer [18, 19]. However, Cha and Kim have identified that the promotion of resilience among cancer survivors can be easily undermined by the degree of uncertainty [20], suggesting the potential regulatory effect of uncertainty on resilience among cancer patients. Hence, considering the protective function of resilience in the psychological status of cancer survivors, it could be inferred that CRF might be influenced by uncertainty through diminishing psychological resilience.

### Hypothesis 3: self-care significantly mediates the relationship between uncertainty and CRF

Besides psychological resilience, self-care is recognized as another promising factor that positively influences the health outcomes of cancer patients. Self-care is referred to “the process of deliberately engaging in behaviors that promote overall health and well-being” [21]. Although PICC catheterization is a feasible method to minimize trauma and enable prolonged use, inadequate self-care post-discharge significantly elevates the risks of infection, arm swelling, and catheter-related venous thrombosis. These complications are closely associated with poor prognosis in BC patients and could pose life-threatening consequences if untreated [22]. Therefore, it requires a high quality of self-management ability and professional nursing care interventions to mitigate PICC chemotherapy-related complications. However, a cross-sectional study revealed that BC patients with higher levels of uncertainty tend to exhibit suboptimal self-care practices when managing the side effects of cancer chemotherapy [14]. Furthermore, a previous multicenter survey conducted by O’Regan et al. demonstrated that the decreased self-care abilities in primary cancer patients undergoing chemotherapy is significantly associated with the onset of CRF, and implementation of self-care strategies has shown potential to alleviate CRF levels among these survivors [23]. Therefore, we assumed that self-care might mediate the impact of uncertainty on CRF among BC patients undergoing PICC chemotherapy.

### Hypothesis 4: psychological resilience and self-care jointly mediate the relationship between uncertainty and CRF

In regard to the relationship between psychological resilience and self-care, a meta-analysis has highlighted a significant correlation between a high level of resilience and the appropriate performance of self-care among patients dealing with chronic illness [24]. Additionally, Tam et al. have proposed a positive link between resilience and self-care behaviors, indicating that resilience plays a crucial role in safeguarding the psychological health of Chinese college students amidst the challenges posed by the COVID-19 pandemic [25]. Similarly, Chang et al. have emphasized that resilience ameliorates depressive symptoms in heart failure patients by bolstering their confidence in self-care practices and consistently adhering to them [26]. This implies that individuals like cancer survivors, when confronted with uncertain conditions, are able to demonstrate appropriate self-care behaviors due to their heightened sense of resilience and self-assurance [27]. This evidence hinted at a potential strong correlation between psychological resilience and self-care, suggesting that both factors might jointly act as mediators in the relationship between uncertainty and CRF among cancer survivors. Specifically, we supposed that BC patients with high uncertainty might struggle to cultivate the necessary psychological resilience to effectively manage issues and stressors stemming from PICC chemotherapy, consequently exacerbating their fatigue levels.

**Fig. 1:**
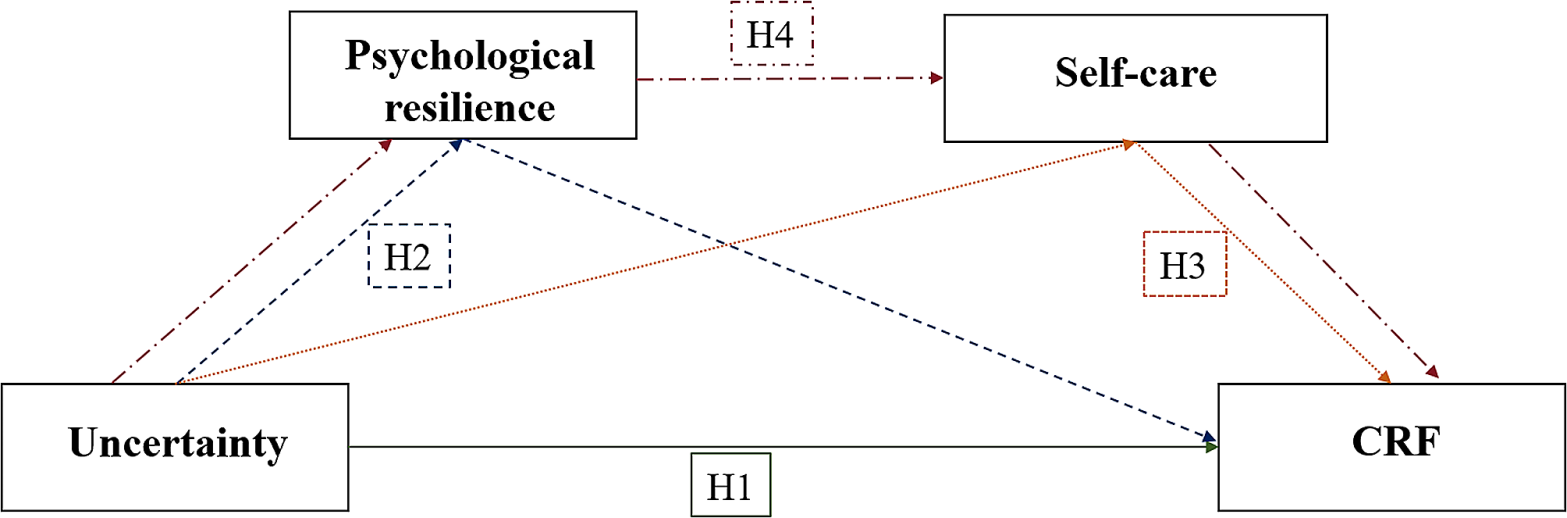
Proposed theoretical model. H1, uncertainty affects CRF; H2, psychological resilience mediates the relationship between uncertainty and CRF; H3, self-care mediates the relationship between uncertainty and CRF; H4, psychological resilience and self-care play chain intermediary in the relationship between uncertainty and CRF. CRF: cancer-related fatigue; H: hypothesis

There has been a growing body of research focusing on the physical and psychological health of women with BC, however, the correlation between uncertainty, resilience, self-care, and CRF in BC patients undergoing PICC chemotherapy has been rarely reported. Hence, in order to investigate the mediating impact of uncertainty on CRF and offer insights for healthcare professionals to develop targeted interventions to alleviate CRF in BC patients undergoing PICC treatment, a theoretical model was conducted based on aforementioned evidence and the four hypotheses proposed in this study (see Fig. 1).

## Methods

### Study design and participant recruitment

A cross-sectional design study was conducted from February 2021 to December 2022 at two tertiary affiliated hospitals of China Medical University in Liaoning, China. A convenient sample of 240 BC cases undergoing PICC chemotherapy was recruited according to the following inclusion criteria: (1) female individuals aged > 18 years old; (2) newly diagnosed with BC; (3) currently receiving PICC chemotherapy; (4) signed informed consent. The exclusion criteria were: (1) had cognitive, communicating, or reading difficulties; (2) psychiatric disorders; (3) other types of cancer or life-threatening diseases; (3) severe infection at the puncture site, significant coagulation abnormalities, or immunodeficiency (4) BC recurrence or metastasis.

### Sample size

The sample size was calculated to be 5 to 10 times the number of variables following Kendall’s principle [28]. Given that our study included 22 variables (11 demographic variables and 11 measurement subscales), a minimum of 138 participants was needed, accounting for a 20% dropout rate. A total of 240 BC patients were recruited for this study. Eventually, 223 valid questionnaires were collected, yielding a valid response of 92.92%.

### Ethical statement

This study received approval from the Ethics Committee of The First Affiliated Hospital of China Medical University (Ethical No. EC-2021-HS-016) and in accordance with the principles of the Declaration of Helsinki. Written informed consent was obtained from all participants, and this study strictly adhered to the STROBE Statement.

### Data collection

Before the survey, the contents and purpose of this study were concisely explained to all participants. Subsequently, our trained researchers distributed the Chinese version of the self-reporting survey questionnaires to each participant independently. It took approximately 15 to 20 min to complete the survey. Besides providing necessary explanation to participants regarding any confusing items, it was strictly prohibited to offer any incentives or misguidance to minimize self-reported bias. Additionally, participants were assured that the data collected from their response would be kept confidential, anonymous, and solely used for scientific research purposes.

### Measurements

#### Sociodemographic and clinical information collection

The patients’ socio-demographic characteristics including age, education, employment, marital status, whether they have children, and monthly income were collected by using a demographic questionnaire. The clinical data on BC duration, the site of PICC placement, the punctured vein, cancer stage, treatment type, and chemotherapy cycle length were obtained from their medical records.

#### Cancer-related uncertainty

Cancer-related uncertainty was assessed using the Chinese version of Mishel Uncertainty in Illness Scale for Adults (MUIS-A) [29]. It consisted of 25 items categorizing into two dimensions: ambiguity (15 items) and complexity (10 items). Each item was ranked on a 5-point Likert scale ranging from 1 (totally disagree) to 5 (strongly agree). The total score ranged from 25 to 125, with higher scores representing higher levels of uncertainty. A score of 25–57 was identified as low level of uncertainty; 58–91 as moderate level of uncertainty; 92–125 as high level of uncertainty. The Cronbach’s α coefficient of the MUIS-A in this study was 0.95.

#### Psychological resilience

Psychological resilience was measured using the Chinese version of Connor-Davidson resilience scale (CD-RISC) [30]. This 25-item scale included three domains: tenacity (13 items), strength (eight items), and optimization (four items) on a 5-point Likert method. Each item was scored based on how closely it matched the participant’s actual circumstances, ranging from 0 (never) to 4 (always). The total score was 100, with higher scores indicating stronger resilience. The Cronbach’s α coefficient of the CD-RISC in this study was 0.89.

#### Self-care

The appraisal of Self-Care Agency Scale-Revised (ASAS-R) scale was employed to measure the self-care capacity of the participants [31]. It consisted of 15 items and was divided into three dimensions to assess an individual’s general self-care capacity (six items), developmental self-care capacity (five items), and lack of self-care capacity (four items). A 5-point Likert-type scale ranging from 1 (strongly disagree) to 5 (strongly agree) was utilized, resulting in a total score range of 15 to 75 points. Higher scores implied better self-care capacities. The Cronbach’s α coefficient of the ASAS-R in this study was 0.88.

#### Cancer-related fatigue (CRF)

CRF was measured using the Chinese version of Cancer Fatigue Scale (CFS-C) [32], which consisted of 15 items covering three dimensions: physical fatigue (seven items), cognitive fatigue (four items), and emotional fatigue (four items). Each item was scored on a 5-point Likert-type scale ranging from 0 (no fatigue) to 4 (severe fatigue). The total score was 60, with higher CFS scores indicating greater severities of fatigue. The Cronbach’s α coefficient of the CFS-C in this study was 0.89.

### Statistical analyses

All data analysis was performed using IBM SPSS Statistics 25.0 software (IBM Corp., NY, USA). Descriptive statistics were used to measure the demographic and clinical variables of the respondents. Continuous data was presented as mean ± standard deviation (SD), while categorical data was expressed as frequency (%). The independent student’s *t*-test and one-way analysis of variance test were used for univariate analysis. Pearson correlation coefficient (r) was employed to examine the relationship between resilience, self-care, uncertainty, and CRF. Education level, cancer duration, cancer stage, and PICC chemotherapy length that had significant effects on CRF were controlled as covariate variables. A hierarchical multiple linear regression analysis was performed to detect the potential variables influencing CRF. The variance inflation factor (VIF) of each variable < 5 was considered no multicollinearity exist [33] For the mediation analysis, a structural equation model (SEM) was executed using Amos 24.0 software (IBM Corp., NY, USA). Fitting indexes were evaluated based on the following criteria: chi-square degrees of freedom (χ2/df) < 3, comparative fix index (CFI) > 0.90, goodness of fit index (GFI) > 0.90, adjusted goodness of fit index (AGFI) > 0.90, Tucker-Lewis index (TLI) > 0.90, standardized root mean square residual (SRMR) < 0.08, and the root mean square error of approximation (RMSEA) < 0.08 [34]. A Bootstrapping method with 5000 replicated samples and a 95% Confidence Interval (CI) was used to determine the significance of the mediating effect. It was supposed that the 95% CI of the mediation path did not contain zero, representing a statistically significant mediating effect. A *p*-value < 0.05 (two-sided) indicated statistical significance.

## Results

### Common method bias test

Harman’s single-factor test was employed to determine the presence of common methodological bias in the collected data. The results revealed that there were 20 factors with eigenvalues greater than 1. The first factor explained 31.28% of the total variance, which was below the recommended standard of 40%. Thus, there was no significant common method bias in this study.

### Participants characteristics

Among these 223 participants, the average age was 52.39 ± 11.08, ranging from 32 to 72 years old. Other demographic information was shown in Table 1. Meanwhile, the univariate analysis revealed that lower educational level (*p* = 0.024), advanced cancer stage (*p* = 0.019), cancer duration (*p* < 0.001), and longer chemotherapy cycle (*p* = 0.032) were significantly correlated the higher scores of CRF (Table 1).

**Table 1:** Demographic and clinical characteristics of participants (*n* = 223)

Characteristics	Categories	No. (%)	CRF score (mean ± SD)	F/t-value	*p*-value
Age (years)	< 40	46 (20.62)	38.63 ± 7.26	0.861^b^	0.424
	40–60	104 (46.64)	37.39 ± 8.05		
	61–72	73 (32.74)	36.81 ± 6.49		
Marital status	Unmarried	35 (15.70)	38.37 ± 7.56	0.795^c^	0.428
	Married	188 (84.30)	37.29 ± 7.38		
Education level	Below high school	127 (56.95)	38.46 ± 6.57	2.341^c^	0.024*
	High school or above	96 (43.05)	36.14 ± 8.23		
Have children	Yes	189 (84.75)	37.37 ± 7.16	0.438^c^	0.662
	No	34 (15.25)	37.97 ± 8.74		
Monthly income	< 3500 yuan	134 (60.09)	38.07 ± 7.05	1.532^c^	0.127
	≥ 3500 yuan	89 (39.91)	36.53 ± 7.85		
Cancer stage	I-II	140 (62.78)	36.56 ± 7.43	2.363^c^	0.019*
	III-IV	83 (37.22)	38.96 ± 7.15		
PICC placement site	Left upper limb	175 (78.48)	37.18 ± 7.43	1.080^c^	0.281
	Right upper limb	48 (21.52)	38.48 ± 7.30		
Punctured vein	Basilic vein	106 (47.53)	37.80 ± 7.51	0.219^b^	0.804
	Cephalic vein	90 (40.36)	37.17 ± 7.29		
	Median cubital vein	27 (12.11)	37.07 ± 7.62		
Cancer duration	≤ 6 months	57 (25.56)	34.00 ± 6.74	4.239^c^	< 0.001***
	> 6 months	166 (74.44)	38.64 ± 7.27		
Treatment type	Chemotherapy	31 (13.90)	36.35 ± 7.81	0.893^c^	0.373
	Chemotherapy and other^a^	192 (86.10)	37.64 ± 7.34		
Chemotherapy length	≤ 4 cycle	177 (79.37)	36.91 ± 7.30	2.162^c^	0.032*
	> 4 cycle	46 (20.63)	39.54 ± 7.50		

Note. CRF, cancer-related fatigue; PICC, peripherally inserted central catheter; SD, standard deviation. ^a^Surgery, radiotherapy, immunotherapy, hormonal therapy; ^b^One-way analysis of variance; ^c^Student’s *t*-test. *p < 0.05, ***p < 0.001

### Correlations between resilience, self-care, uncertainty, and CRF

Our statistical results showed that the average total scores of CRF, uncertainty, psychological resilience, and self-care were 37.46 ± 7.40, 66.82 ± 11.48, 42.31 ± 9.92, and 35.35 ± 6.97, respectively, among the 223 BC patients undergoing PICC chemotherapy (Table 2). Pearson correlation analysis revealed that uncertainty was positively related to CRF (*r* = 0.749, *p* < 0.01), while negatively correlated with psychological resilience (*r* = -0.649, *p* < 0.01) and self-care (*r* = -0.704, *p* < 0.01); Psychological resilience was positively correlated with self-care (*r* = 0.555, *p* < 0.01), but negatively correlated with CRF (*r* = -0.722, *p* < 0.01). Moreover, a negative correlation was found between self-care and CRF (*r* = -0.675, *p* < 0.01, Table 2).

**Table 2:** Pearson’s correlation coefficients of variables in the participants (*n* = 223)

Variables	Mean	SD	1.	2.	3.	4.	5.	6.	7.	8.	9.
1.Uncertainty	66.82	11.48	1								
2.Ambiguity	41.52	8.50	0.918**	1							
3.Complexity	27.32	6.46	0.877**	0.879**	1						
4.PR	42.31	9.92	-0.649**	-0.633**	-0.627**	1					
5.Self-care	35.35	6.97	-0.704**	-0.652**	-0.592**	0.555**	1				
6.CRF	37.46	7.40	0.749**	0.767**	0.735**	-0.722**	-0.675**	1			
7.Physical fatigue	17.53	3.44	0.700**	0.719**	0.678**	-0.690**	-0.637**	0.945**	1		
8.Cognitive fatigue	9.83	2.24	0.674**	0.697**	0.693**	-0.650**	-0.610**	0.879**	0.739**	1	
9.Emotional fatigue	10.09	2.40	0.679**	0.686**	0.652**	-0.632**	-0.602**	0.911**	0.795**	0.722**	1

Note. **p < 0.01. CRF, cancer-related fatigue; PR, psychological resilience; SD, standard deviation

### Hierarchical regression analysis

The issue of multicollinearity bias was excluded by examining the VIF values, which ranged from 1.050 to 2.486 (< 5) in this study. Table 3 presented the results of the hierarchical multiple regression analysis. In Model 1, with education level, cancer duration, cancer stage, and PICC chemotherapy length as covariates, the explanatory power of these covariate variables was 4.90% [F (4, 218) = 3.878, *p* = 0.005]. Upon introducing uncertainty in Model 2, we found that uncertainty was positively predicted CRF (β = 0.729, *p* < 0.001), explaining 56.30% of the variability in CRF [F (1, 217) = 58.274, *p* < 0.001]. Subsequently, in Model 3, the impact of uncertainty on CRF reduced from 0.729 to 0.474, with the inclusion of psychological resilience, which inversely influenced CRF (β = -0.399, *p* < 0.001). The variables in Model 3 collectively explained 65.50% of the variance [F (1, 216) = 71.203, *p* < 0.001]. Furthermore, incorporating self-care in Model 4 led to a decrease in the effects of uncertainty and psychological resilience on CRF to 0.353 and − 0.366, respectively. Moreover, self-care (β = -0.208, *p* < 0.001) were found to be the negative predictor of CRF, contributing to an additional explanatory power of 2.00% [F (1, 215) = 66.567, *p* < 0.001]. These findings implied the potential chain mediating role of psychological resilience and self-care in linking uncertainty to CRF.

**Table 3:** Hierarchical multiple regression analysis of influencing variables of CRF

Variables	Model 1			Model 2			Model 3				Model 4		
	B	β	*t*	B	β	*t*	B		β	*t*	B	β	*t*
Constants	35.580		24.821***	5.458		2.583*	29.135			8.049***	41.024		8.608***
Education	-1.851	-0.124	-1.839	-0.539	-0.036	-0.785	-0.411		-0.028	-0.673	-0.270	-0.018	-0.453
Duration	0.153	0.080	1.179	0.010	0.005	0.116	0.014		0.007	0.177	0.031	0.016	0.400
Cancer stage	1.868	0.122	1.800	1.358	0.089	1.929	0.957		0.063	1.524	0.740	0.048	1.207
PICC length	2.311	0.127	1.879	0.893	0.049	1.065	0.889		0.049	1.194	0.411	0.023	0.559
Uncertainty				0.471	0.729	16.050***	0.306		0.474	9.032***	0.228	0.353	5.850***
PR							-0.298		-0.399	-7.653***	-0.273	-0.366	-7.109***
Self-care											-0.221	-0.208	-3.698***
R^2^	0.066			0.573			0.664				0.684		
adjust R^2^	0.049			0.563			0.655				0.674		
F	F (4, 218) = 3.878**			F (1, 217) = 58.274***			F (1, 216) = 71.203***				F (1, 215) = 66.567***		
ΔR^2^	0.066			0.507			0.091				0.020		
ΔF	3.878			257.594			58.561				13.676		

Note. *p < 0.05; **p < 0.01; ***p < 0.001. CRF, cancer-related fatigue; PR, psychological resilience

### Test for the mediation model

**Fig. 2:**
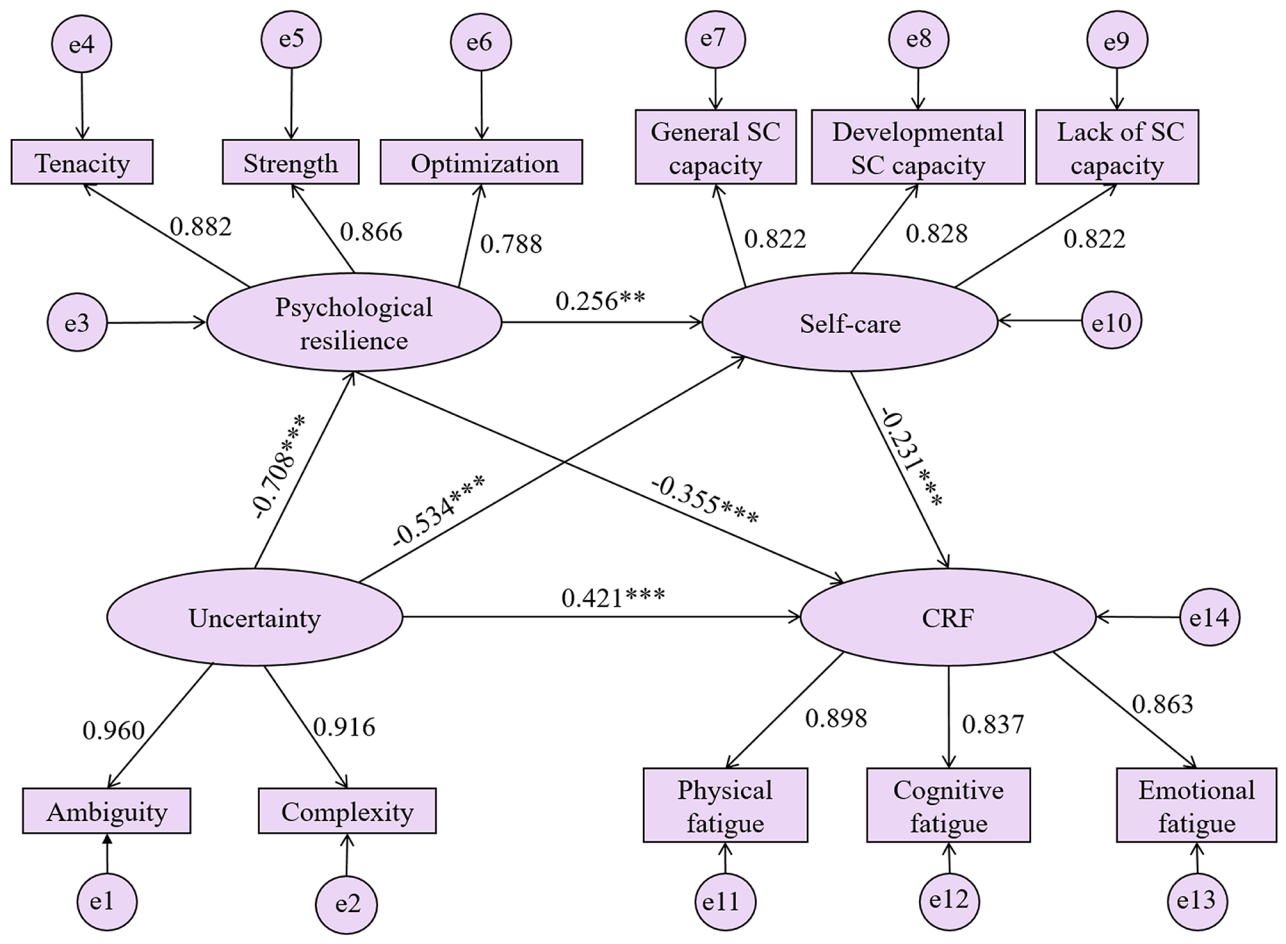
Structural equation modeling of proposed theoretical model. Values on Paths were standardized coefficients. **p < 0.01, ***p < 0.001. CRF: cancer-related fatigue; SC: self-care

The established SEM demonstrated favorable fit indices: χ2/df = 1.408 (< 3), CFI = 0.983 (> 0.90), GFI = 0.933 (> 0.90), AGFI = 0.904 (> 0.90), TLI = 0.978 (> 0.90), SRMR = 0.079 (< 0.08), and RMSEA = 0.043 (< 0.08), indicating the model fitted well. Standardized coefficients in Fig. 2 revealed statistically significant relationships for all paths.

The Bootstrap test method was performed to assess the significance of each mediating path (see Table 4). The findings revealed that the direct effect of uncertainty on CRF was 0.146, accounting for 50.17% of the total effect, with a 95% CI of 0.071 to 0.215 and *p* = 0.001, implying statistically significant (Table 4). Subsequently, by adding psychological resilience and self-care as mediating variables, the total indirect effect of uncertainty on CRF accounted for 49.83% of the total effect (95% CI = 0.086 to 0.223, *p* = 0.001). Three mediating paths were identified: Path 1: Uncertainty → psychological resilience → CRF, with a mediating effect of 0.087 and a 95% CI (0.035 to 0.161), accounting for 29.90% of the total effect; Path 2: Uncertainty → self-care → CRF, showing a mediating effect of 0.043 and a 95% CI (0.003 to 0.128), contributing to 14.78% of the total effect; Path 3: Uncertainty → psychological resilience → self-care → CRF, displaying a mediating effect was 0.015 and a 95% CI (0.002 to 0.042), with an effect ratio of 5.15% (Table 4). Notably, the 95% CI for all three paths did not contain zero, suggesting a sequential mediating effect of psychological resilience and self-care in the relationship between uncertainty and CRF.

**Table 4:** Significance test of Bootstrap mediating effect

Paths	Effect	BootSE	Boot 95% CI		*p*-value	Effect size
			Lower	Upper		
Total effect	0.291	0.023	0.250	0.339	< 0.001	
Direct effect	0.146	0.038	0.071	0.215	0.001	50.17%
Indirect effect	0.145	0.037	0.086	0.223	0.001	49.83%
Path1: uncertainty→PR→CRF	0.087	0.033	0.035	0.161	0.001	29.90%
Path2: uncertainty→self-care→CRF	0.043	0.032	0.003	0.128	0.030	14.78%
Path3: uncertainty→PR→self-care→CRF	0.015	0.009	0.002	0.042	0.028	5.15%

Note. CRF, cancer-related fatigue; PR, psychological resilience

## Discussion

PICC implantation is typically performed in the anterior basilica vein in the forearm and remains in place for a minimum of several months. Consequently, the extended catheter retention poses a significant challenge for a considerable number of BC patients. Previous studies have established a negative correlation between PICC chemotherapy-related complications and patients’ health-promoting behaviors and self-management abilities [35]. Thus, it is imperative to identify the influential factors contributing to PICC chemotherapy-associated side effects in BC patients and develop targeted intervention strategies to help them effectively manage PICC chemotherapy independently. To the best of our knowledge, this study was the first cross-sectional investigation to examine the association between uncertainty, psychological resilience, self-care, and CRF among BC patients undergoing PICC chemotherapy. In the present study, we found that uncertainty significantly and positively predicted CRF. Additionally, we found that psychological resilience and self-care played a chain mediating role between uncertainty and CRF. This suggested that higher levels of uncertainty among BC patients undergoing PICC chemotherapy impeded the development of psychological resilience and hindered the improvement of self-care practices, ultimately exacerbating CRF. In brief, our findings provide partial insight into the mechanism through which uncertainty influenced CRF in BC patients undergoing PICC chemotherapy.

### Relationship between uncertainty and CRF

Our data analysis showed that uncertainty significantly influenced and positively predicted CRF in BC patients undergoing PICC chemotherapy. This finding supported Hypothesis 1. In this study, the correlation between uncertainty and CRF suggested that BC patients experiencing higher levels of uncertainty were more likely to trigger adverse emotions such as anxiety, distress, and depression. These emotions could lead to a lack of effective coping strategies and the courage needed to confront long-term PICC chemotherapy, ultimately resulting in self-doubt and a sense of inner fatigue. This observation aligned with an observational cross-sectional study which substantiated a strong association between high levels of fatigue and uncertainty in BC survivors [36]. Meanwhile, Hall et al. also highlighted a significant correlation between heightened cancer-related uncertainty and increased fatigue in younger BC survivors [37]. Additionally, Mast illustrated that fatigue was clearly associated with the chemotherapy treatment method in BC patients, and the variation in uncertainty was significantly attributed to fatigue and education levels [15]. Therefore, given the detrimental impact of uncertainty on the CRF experienced by BC survivors undergoing PICC chemotherapy, healthcare professionals need to implement targeted interventions to address CRF issues engendered by uncertainty. For example, a systematic review conducted by Tuominen et al. found that educational and psychosocial nursing interventions had significant effects on reducing of anxiety, distress, and CRF among cancer patients [38]. Dolgoy et al. also emphasized uncertainty as a novel target for intervention aimed at mitigating the negative influences of CRF on the psychological well-being of cancer patients [10].

### The mediating role of psychological resilience

Our results also revealed that psychological resilience mediated the positive impact of uncertainty on CRF in BC patients undergoing PICC chemotherapy, thus confirming Hypothesis 2. It was worth noting that the high levels of uncertainty experienced by BC patients were prone to have detrimental impacts not only on their physical and psychological health outcomes, but also on their psychological resilience. This could make it difficult for them to adapt to chronic PICC chemotherapy, ultimately exacerbating the severity of CRF. Öcalan and Özcetin strengthened that the negative effects of CRF during the cancer process could be effectively managed by the presence of psychological resilience [39]. Meanwhile, a randomized trail constructed by Lin et al. declared that the enhancement of psychological resilience through attention and interpretation therapy intervention could decrease the level of CRF in patients after colon cancer surgery [18]. Zhang, in a prospective randomized trial study, also elaborated that the implementation of the Knowledge, Attitude, and Practice (KAP) model could improve resilience and reduce CRF in colorectal cancer patients undergoing chemotherapy [40]. Similarly, our study identified a negative correlation between psychological resilience and CRF, providing evidence to support the role of psychological resilience in alleviating CRF and shedding light on how uncertainty affected CRF in BC patients undergoing PICC chemotherapy.

### The mediating role of self-care

It has been reported that proper daily care of PICCs is strongly associated with the physiological and mental health of cancer survivors, leading to the prevention of adverse effects from chemotherapy. However, it appears that cancer patients face challenges in taking care of themselves during PICC chemotherapy due to their limited self-care abilities [41]. It is noted that the self-care is engaged in patients’ health promotion lifestyles, which plays a crucial role in progressively strengthening their attitudes towards self-care behaviors and then effectively enhancing their self-health-promoting capacities [21]. Thus, it is important to prioritize the potential factors influencing engagement in self-health-promoting activities during PICC catheterization. In our study, we found that self-care played a mediating role between uncertainty and CRF, thus confirming Hypothesis 3. Uncertainty could impede BC survivors from accessing self-care skills and hinder their commitment to daily PICC management. Moreover, a lack of self-care abilities during PICC maintenance can prevent survivors from adopting effective coping strategies, leading to increased complications and emotional distress. These findings were in line with the research conducted by Zhang et al. [14]. , who also found that uncertainty could independently predict self-care behaviors among Chinese BC women undergoing chemotherapy. Additionally, a clinical trial study conducted by Williams et al. [42] suggested that reinforcing patient education on proper self-care behavior management effectively mitigated fatigue and anxiety side effects of chemotherapy in BC patients. Accordingly, to improve the well-being of cancer patients during chemotherapy, it is essential to provide comprehensive guidance and education on self-care strategies to effectively manage fatigue [43].

### The chain mediating role of psychological resilience and self-care

Additionally, our findings also demonstrated that psychological resilience and self-care played a sequential mediating role between uncertainty and CRF, thus verifying Hypothesis 4. One possible explanation for this finding was that when BC patients faced uncertainty resulting from long-term PICC chemotherapy, a high level of psychological resilience could help them quickly adapt to these challenges enabling them to make positive adjustments, adopt problem-solving strategies, and actively engage in appropriate self-care behaviors. Consequently, individuals were more likely to alleviate the side effects of CRF, ultimately leading to improving their quality of life. Similarly, Abdollahi et al. also unraveled that resilience was positively correlated with self-care activities in BC patients, highlighting that resilience directly contributed to increased health-related behaviors, thereby improving patients’ quality of life [27]. In the current study the chain mediation effect model of psychological resilience and self-care suggested that health practitioners could effectively diminish CRF in BC patients undergoing PICC chemotherapy by enhancing patients’ psychological resilience while addressing their uncertainty level. Notably, a clinical trial conducted by Mollaei et al. revealed that a well-designed self-care education intervention could boost the resilience of cancer patients [44]. This implied that psychological resilience might be inversely influenced by self-care practices in BC patients. Thus, further research is necessary to explore the bidirectional interaction between psychological resilience and self-care in relation to uncertainty and CRF.

### Practice implications

This study deeply explored the relationship between uncertainty and CRF from a positive mental changes perspective. It was demonstrated that psychological resilience and self-care played pivotal roles in buffering uncertainty, which provides valuable insights for healthcare practitioners to tailor interventions in the future. For instance, healthcare providers could incorporate psychological interventions into BC therapy sessions to equip more patients with effective skills for managing distresssuch as cognitive-behavioral interventions that have already been implemented in clinical settings to help cancer survivors recognize their negative thoughts and fostering positive health-related behaviors. In addition, interventions that support coping strategies, provide education, and involve activities can not only enhance patients’ self-care abilities but also alleviate stress and reduce CRF in BC patients [45]. Therefore, healthcare professionals should attempt to take more comprehensive interventions that address both psychological resilience and self-care ability, as these dual approaches may synergistically mitigate the adverse impact of uncertainty on CRF in BC patients undergoing PICC chemotherapy. This integrated approach could lead to more effective outcomes and an improved quality of life for BC patients.

### Limitations

There were some limitations in this study. Firstly, since this was a cross-sectional study that only demonstrated the correlation between the variables being investigated, it is inevitable to conduct a longitudinal study with an interventional approach in the future to explore the causal relationships among these variables. Secondly, the participants were only recruited from urban hospitals in Shenyang city, which may limit the generalizability of the results. Subsequent studies should involve a broader sample of BC patients from various cities and regions in China to enhance the generalizability of the findings. Thirdly, the data presented were solely obtained from self-reported surveys completed by the participants, which might involve subjective bias as it relied on the participants’ own perceptions and feelings. Furthermore, the COVID-19 pandemic might also have an impact on their emotional status. Therefore, further studies need to be conducted to thoroughly validate the effects of these variables in BC patients undergoing PICC chemotherapy.

### Conclusions

In summary, the current study demonstrated that uncertainty could not only positively and directly predict CRF in BC patients undergoing PICC chemotherapy, but also indirectly predict CRF through psychological resilience and self-care, as well as the chain mediating effect between psychological resilience and self-care. Given the beneficial impact of psychological resilience and self-care in alleviating CRF in BC patients undergoing PICC chemotherapy, it is crucial to provide effective interventions to a larger cohort of BC patients to assist them in cultivating stronger psychological resilience and enhancing their self-care abilities, especially in dealing with uncertainty and mitigating CRF.

## Data Availability

The data that support the findings of this study are available from the corresponding author upon reasonable request.

## Abbreviations

95% CI: 95% Confidence interval
AGFI: Adjusted goodness of fit index
BC: Breast cancer
CRF: Cancer-related fatigue
CFI: Comparative fix index
GFI: Goodness of fit index
PICC: Peripherally inserted central catheter
RMSEA: Root mean square error of approximation
SD: Standard deviation
SEM: Structural equation model
SRMR: Standardized root mean square residual
VIF: Variance inflation factor
χ2/df: Chi-square degrees of freedom
